# *N*′-[(*E*)-4-Bromo­benzyl­idene]-2-(4-isobutyl­phen­yl)propanohydrazide

**DOI:** 10.1107/S1600536809015542

**Published:** 2009-05-07

**Authors:** Hoong-Kun Fun, Ching Kheng Quah, K. V. Sujith, B. Kalluraya

**Affiliations:** a X-ray Crystallography Unit, School of Physics, Universiti Sains Malaysia, 11800 USM, Penang, Malaysia; b Department of Studies in Chemistry, Mangalore University, Mangalagangotri, Mangalore 574 199, India

## Abstract

The asymmetric unit of the title compound, C_20_H_23_BrN_2_O, contains two independent mol­ecules (*A* and *B*), in which the orientations of the 4-isobutyl­phenyl units are different. The dihedral angle between the two benzene rings is 88.45 (8)° in mol­ecule *A* and 89.87 (8)° in mol­ecule *B*. Mol­ecules *A* and *B* are linked by a C—H⋯N hydrogen bond. In the crystal, mol­ecules are linked into chains running along the *a* axis by inter­molcular N—H⋯O and C—H⋯O hydrogen bonds. The crystal structure is further stabilized by C—H⋯π inter­actions. The presence of pseudosymmetry in the structure suggests the higher symmetry space group *Pbca*. However, attempts to refine the structure in this space group resulted in a disorder model with high *R* (0.097) and *wR* (0.257) values. The crystal studied was an inversion twin with a 0.595 (4):0.405 (4) domain ratio.

## Related literature

For the biological activities of hydrazone derivatives, see: Bedia *et al.* (2006 ▶); Rollas *et al.* (2002 ▶); Terzioglu & Gürsoy (2003 ▶); Sridhar & Perumal (2003 ▶); Amir & Kumar (2007 ▶). For a related structure, see: Fun *et al.* (2008 ▶). For bond-length data, see: Allen *et al.* (1987 ▶). For the stability of the temperature controller used for the data collection, see: Cosier & Glazer (1986 ▶).
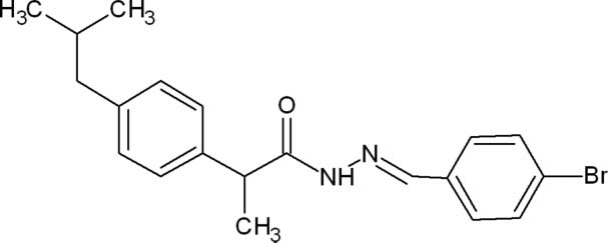


## Experimental

### 

#### Crystal data

C_20_H_23_BrN_2_O
*M* *_r_* = 387.31
Orthorhombic, 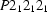

*a* = 9.1440 (1) Å
*b* = 12.0110 (1) Å
*c* = 33.5670 (4) Å
*V* = 3686.62 (7) Å^3^
*Z* = 8
Mo *K*α radiation
μ = 2.24 mm^−1^
*T* = 100 K
0.49 × 0.38 × 0.19 mm

#### Data collection

Bruker SMART APEXII CCD area-detector diffractometer
Absorption correction: multi-scan (**SADABS**; Bruker, 2005 ▶) *T*_min_ = 0.407, *T*_max_ = 0.682
120355 measured reflections
20557 independent reflections
13317 reflections with *I* > 2σ(*I*)
*R*_int_ = 0.045

#### Refinement

*R*[*F* ^2^ > 2σ(*F* ^2^)] = 0.042
*wR*(*F* ^2^) = 0.103
*S* = 1.04
20557 reflections
448 parameters
H atoms treated by a mixture of independent and constrained refinement
Δρ_max_ = 0.72 e Å^−3^
Δρ_min_ = −0.62 e Å^−3^
Absolute structure: Flack (1983 ▶), 9205 Friedel pairs
Flack parameter: 0.595 (4)

### 

Data collection: *APEX2* (Bruker, 2005 ▶); cell refinement: *SAINT* (Bruker, 2005 ▶); data reduction: *SAINT*; program(s) used to solve structure: *SHELXTL* (Sheldrick, 2008 ▶); program(s) used to refine structure: *SHELXTL*; molecular graphics: *SHELXTL*; software used to prepare material for publication: *SHELXTL* and *PLATON* (Spek, 2009 ▶).

## Supplementary Material

Crystal structure: contains datablocks global, I. DOI: 10.1107/S1600536809015542/ci2787sup1.cif

Structure factors: contains datablocks I. DOI: 10.1107/S1600536809015542/ci2787Isup2.hkl

Additional supplementary materials:  crystallographic information; 3D view; checkCIF report

## Figures and Tables

**Table 1:** Selected torsion angles (°)

C8*A*—C9*A*—C10*A*—C11*A*	−96.54 (17)
C8*A*—C9*A*—C10*A*—C15*A*	80.18 (18)
C20*A*—C9*A*—C10*A*—C11*A*	141.61 (15)
C20*A*—C9*A*—C10*A*—C15*A*	−41.7 (2)
C8*B*—C9*B*—C10*B*—C11*B*	77.53 (17)
C8*B*—C9*B*—C10*B*—C15*B*	−101.83 (16)
C20*B*—C9*B*—C10*B*—C11*B*	−160.06 (14)
C20*B*—C9*B*—C10*B*—C15*B*	20.6 (2)

**Table 2:** Hydrogen-bond geometry (Å, °)

*D*—H⋯*A*	*D*—H	H⋯*A*	*D*⋯*A*	*D*—H⋯*A*
N2*A*—H1*NA*⋯O1*B*^i^	0.91 (2)	1.97 (2)	2.830 (2)	157 (2)
N2*B*—H1*NB*⋯O1*A*^ii^	0.77 (2)	2.11 (2)	2.826 (2)	155 (2)
C7*A*—H7*AA*⋯O1*B*^i^	0.93	2.45	3.241 (2)	143
C7*B*—H7*BA*⋯O1*A*^ii^	0.93	2.53	3.307 (3)	141
C20*B*—H20*F*⋯N1*A*	0.96	2.56	3.494 (2)	164
C12*A*—H12*A*⋯*Cg*2^i^	0.93	2.66	3.482 (2)	148
C12*B*—H12*B*⋯*Cg*1^ii^	0.93	2.79	3.680 (2)	160

Symmetry codes: (i) 
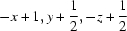
; (ii) 
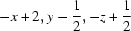
. *Cg*1 and *Cg*2 are the centroids of the C1*A*–C6*A* and C1*B*–C6*B* benzene rings, respectively.

**Fig. 1.**
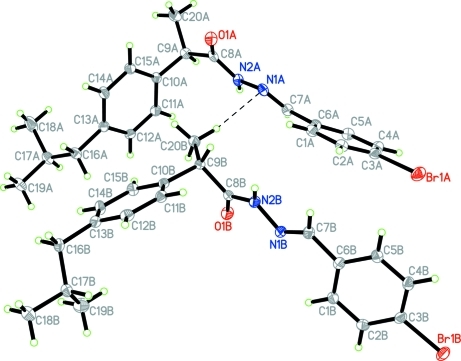
The molecular structure of the title compound, showing 50% probability displacement ellipsoids and the atom-numbering scheme. The dashed line indicates a hydrogen bond.

**Fig. 2.**
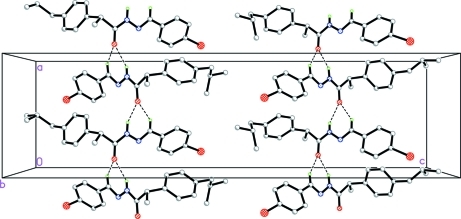
The crystal packing of the title compound, viewed along the a axis. Intermolecular interactions are shown as dashed lines.

## supplementary crystallographic information

### Comment

Hydrazones have been found to possess antimicrobial, anticonvulsant, analgesic, antiinflammatory, antiplatelet, antitubercular, anticancer and antitumoral activities (Bedia *et al.*, 2006; Rollas *et al.*, 2002; Terzioglu & Gürsoy, 2003). Aryl hydrazones are important building blocks for the synthesis of a variety of heterocyclic compounds such as pyrazolines and pyrazoles (Sridhar *et al.*, 2003). Aryl hydrazones have been most conveniently synthesized by the reaction of aryl hydrazines with carbonyl compounds. Similarly ibuprofen is also known for their pharmaceutical activities and belongs to the class of Non-Steroidal Anti-Inflammatory Drugs (Amir & Kumar, 2007). We are interested in the synthesis and crystal structure of ibuprofen containing hydrazone derivatives (Fun *et al.*, 2008). Prompted by these observations, it was contemplated to synthesize and report the crystal structure of the title compound.

The asymmetric unit contains two independent molecules (Fig. 1), *A* and *B*, in which the orientations of the 4-isobutylphenyl units are different (Table 1). The bond lengths (Allen *et al.*, 1987) and angles in the molecule (Fig. 1) are within normal ranges and are comparable to a closely related structure (Fun *et al.*, 2008). The molecule *A* is linked to the molecule *B* by C20B—H20F···N1A hydrogen bond (Fig. 1). The dihedral angle formed by the C1A-C6A and C10A-C15A benzene rings is 89.87 (8)° and that between the C1B-C6B and C10B-C15B planes is 88.45 (8)°, indicating that they are almost perpendicular to each other.

The crystal packing is consolidated by intermolecular N—H···O and C—H···O hydrogen bonds (Fig. 2) which link the independent molecules into chains parallel to the [100]. The crystal structure is further stabilized by C—H···π interactions (Table 1) involving the C1A-C6A (centroid *Cg*1) and C1B–C6B (centroid *Cg*2) benzene rings.

### Experimental

The title compound was obtained by refluxing a mixture of 2-[4-(2-methylpropyl)phenyl]propanehydrazide (0.01 mol), 4-bromobenzaldehyde (0.01 mol) in ethanol (30 ml) and 3 drops of concentrated sulfuric acid for 1 h. Excess ethanol was removed from the reaction mixture under reduced pressure. The solid product obtained was filtered, washed with ethanol and dried. Single crystals suitable for X-ray analysis were obtained by slow evaporation of an ethanol-*N*,*N*-dimethylformamide (DMF) (3:1) solution.

### Refinement

Atoms H1NA and H1NB were located in a difference difference Fourier map and refined freely. The remaining H atoms were positioned geometrically and refined using a riding model, with C-H = 0.93–0.98 Å and *U*_iso_(H) = 1.2 or 1.5 *U*_eq_(C). A rotating-group model was applied for the methyl groups. The presence of pseudo-symmetry in the structure suggests a higher symmetry space group *Pbca*. But attempts to refine the structure in the space group *Pbca* resulted in a disorder model with high R (0.097) and wR (0.257) values. Because of the presence of a pseudo-centre of symmetry, the absolute structure could not be determined. The reported Flack parameter was obtained by TWIN/BASF procedure in SHELXL (Sheldrick, 2008).

### Crystal data

**Table:**

C_20_H_23_BrN_2_O	*F*(000) = 1600
*M**_r_* = 387.31	*D*_x_ = 1.396 Mg m^−^^3^
Orthorhombic, *P*2_1_2_1_2_1_	Mo *K*α radiation, λ = 0.71073 Å
Hall symbol: P 2ac 2ab	Cell parameters from 9400 reflections
*a* = 9.1440 (1) Å	θ = 2.5–31.7°
*b* = 12.0110 (1) Å	µ = 2.24 mm^−^^1^
*c* = 33.5670 (4) Å	*T* = 100 K
*V* = 3686.62 (7) Å^3^	Block, colourless
*Z* = 8	0.49 × 0.38 × 0.19 mm

### Data collection

**Table:**

Bruker SMART APEXII CCD area-detector diffractometer	20557 independent reflections
Radiation source: fine-focus sealed tube	13317 reflections with *I* > 2σ(*I*)
graphite	*R*_int_ = 0.045
φ and ω scans	θ_max_ = 38.6°, θ_min_ = 1.2°
Absorption correction: multi-scan (*SADABS*; Bruker, 2005)	*h* = −16→16
*T*_min_ = 0.407, *T*_max_ = 0.682	*k* = −21→20
120355 measured reflections	*l* = −58→56

### Refinement

**Table:**

Refinement on *F*^2^	Secondary atom site location: difference Fourier map
Least-squares matrix: full	Hydrogen site location: inferred from neighbouring sites
*R*[*F*^2^ > 2σ(*F*^2^)] = 0.042	H atoms treated by a mixture of independent and constrained refinement
*wR*(*F*^2^) = 0.103	*w* = 1/[σ^2^(*F*_o_^2^) + (0.0498*P*)^2^] where *P* = (*F*_o_^2^ + 2*F*_c_^2^)/3
*S* = 1.04	(Δ/σ)_max_ = 0.001
20557 reflections	Δρ_max_ = 0.72 e Å^−^^3^
448 parameters	Δρ_min_ = −0.61 e Å^−^^3^
0 restraints	Absolute structure: Flack (1983), 9205 Friedel pairs
Primary atom site location: structure-invariant direct methods	Flack parameter: 0.595 (4)

### Special details

**Table:**

Experimental. The crystal was placed in the cold stream of an Oxford Cyrosystems Cobra open-flow nitrogen cryostat (Cosier & Glazer, 1986) operating at 100.0 (1) K.
Geometry. All esds (except the esd in the dihedral angle between two l.s. planes) are estimated using the full covariance matrix. The cell esds are taken into account individually in the estimation of esds in distances, angles and torsion angles; correlations between esds in cell parameters are only used when they are defined by crystal symmetry. An approximate (isotropic) treatment of cell esds is used for estimating esds involving l.s. planes.
Refinement. Refinement of *F*^2^ against ALL reflections. The weighted *R*-factor *wR* and goodness of fit *S* are based on *F*^2^, conventional *R*-factors *R* are based on *F*, with *F* set to zero for negative *F*^2^. The threshold expression of *F*^2^ > σ(*F*^2^) is used only for calculating *R*-factors(gt) *etc*. and is not relevant to the choice of reflections for refinement. *R*-factors based on *F*^2^ are statistically about twice as large as those based on *F*, and *R*- factors based on ALL data will be even larger.

### Fractional atomic coordinates and isotropic or equivalent isotropic displacement parameters (Å^2^)

**Table:**

	*x*	*y*	*z*	*U*_iso_*/*U*_eq_	
Br1A	0.849271 (19)	0.479672 (14)	0.078235 (6)	0.02563 (4)	
O1A	0.88325 (12)	1.00346 (11)	0.27706 (3)	0.0213 (2)	
N1A	0.73139 (15)	0.87330 (12)	0.22421 (4)	0.0173 (3)	
N2A	0.66653 (15)	0.94990 (12)	0.24939 (4)	0.0164 (3)	
C1A	0.81210 (19)	0.67440 (14)	0.17959 (5)	0.0184 (3)	
H1AA	0.8601	0.6813	0.2039	0.022*	
C2A	0.85669 (18)	0.59430 (13)	0.15259 (5)	0.0187 (3)	
H2AA	0.9328	0.5460	0.1589	0.022*	
C3A	0.78674 (19)	0.58675 (14)	0.11603 (5)	0.0189 (3)	
C4A	0.6703 (2)	0.65496 (14)	0.10623 (5)	0.0211 (3)	
H4AA	0.6243	0.6487	0.0816	0.025*	
C5A	0.62366 (19)	0.73315 (14)	0.13401 (5)	0.0192 (3)	
H5AA	0.5437	0.7781	0.1281	0.023*	
C6A	0.6946 (2)	0.74535 (13)	0.17050 (5)	0.0165 (3)	
C7A	0.64467 (19)	0.83222 (13)	0.19787 (5)	0.0176 (3)	
H7AA	0.5490	0.8581	0.1962	0.021*	
C8A	0.74894 (16)	1.00775 (15)	0.27548 (5)	0.0170 (3)	
C9A	0.65950 (18)	1.07453 (13)	0.30584 (5)	0.0172 (3)	
H9AA	0.5624	1.0888	0.2946	0.021*	
C10A	0.64245 (16)	0.99942 (13)	0.34220 (4)	0.0155 (3)	
C11A	0.51587 (18)	0.93583 (14)	0.34705 (5)	0.0185 (3)	
H11A	0.4397	0.9428	0.3289	0.022*	
C12A	0.50265 (19)	0.86228 (13)	0.37874 (5)	0.0182 (3)	
H12A	0.4167	0.8217	0.3817	0.022*	
C13A	0.61539 (18)	0.84797 (13)	0.40622 (4)	0.0172 (3)	
C14A	0.74046 (17)	0.91272 (14)	0.40148 (5)	0.0204 (3)	
H14A	0.8167	0.9056	0.4196	0.025*	
C15A	0.75390 (17)	0.98781 (15)	0.37016 (5)	0.0200 (3)	
H15A	0.8382	1.0307	0.3679	0.024*	
C16A	0.60190 (19)	0.76581 (13)	0.43996 (5)	0.0194 (3)	
H16A	0.6991	0.7508	0.4502	0.023*	
H16B	0.5640	0.6965	0.4293	0.023*	
C17A	0.50438 (19)	0.80147 (14)	0.47485 (5)	0.0219 (3)	
H17A	0.4035	0.8064	0.4652	0.026*	
C18A	0.5468 (3)	0.91335 (16)	0.49144 (6)	0.0442 (6)	
H18A	0.5341	0.9693	0.4713	0.066*	
H18B	0.6473	0.9116	0.4997	0.066*	
H18C	0.4859	0.9305	0.5139	0.066*	
C19A	0.5104 (2)	0.71186 (15)	0.50728 (5)	0.0274 (4)	
H19A	0.4477	0.7328	0.5290	0.041*	
H19B	0.6090	0.7046	0.5168	0.041*	
H19C	0.4784	0.6420	0.4964	0.041*	
C20A	0.73368 (19)	1.18646 (15)	0.31432 (6)	0.0229 (4)	
H20A	0.6876	1.2212	0.3368	0.034*	
H20B	0.7246	1.2339	0.2914	0.034*	
H20C	0.8353	1.1744	0.3200	0.034*	
Br1B	0.63994 (2)	−0.015149 (14)	0.077318 (5)	0.02449 (4)	
O1B	0.61864 (12)	0.49983 (11)	0.27805 (3)	0.0206 (2)	
N1B	0.77028 (15)	0.36554 (11)	0.22662 (4)	0.0161 (3)	
N2B	0.83490 (16)	0.44565 (12)	0.25061 (4)	0.0165 (3)	
C1B	0.68109 (19)	0.17270 (13)	0.18046 (5)	0.0175 (3)	
H1BA	0.6314	0.1809	0.2045	0.021*	
C2B	0.63144 (18)	0.09615 (13)	0.15256 (5)	0.0183 (3)	
H2BA	0.5492	0.0529	0.1577	0.022*	
C3B	0.7075 (2)	0.08541 (13)	0.11670 (5)	0.0190 (3)	
C4B	0.83235 (19)	0.14694 (13)	0.10867 (5)	0.0190 (3)	
H4BA	0.8824	0.1379	0.0848	0.023*	
C5B	0.88101 (19)	0.22226 (13)	0.13705 (5)	0.0180 (3)	
H5BA	0.9654	0.2633	0.1323	0.022*	
C6B	0.8045 (2)	0.23731 (13)	0.17286 (5)	0.0164 (3)	
C7B	0.85619 (19)	0.32373 (13)	0.20041 (5)	0.0175 (3)	
H7BA	0.9524	0.3484	0.1988	0.021*	
C8B	0.75320 (16)	0.50621 (14)	0.27594 (4)	0.0153 (3)	
C9B	0.84083 (18)	0.58372 (12)	0.30309 (4)	0.0144 (3)	
H9BA	0.9371	0.5957	0.2910	0.017*	
C10B	0.86268 (16)	0.52286 (12)	0.34255 (4)	0.0146 (3)	
C11B	0.96862 (18)	0.43906 (13)	0.34546 (5)	0.0165 (3)	
H11B	1.0261	0.4219	0.3234	0.020*	
C12B	0.98872 (19)	0.38154 (13)	0.38080 (5)	0.0188 (3)	
H12B	1.0601	0.3265	0.3821	0.023*	
C13B	0.90454 (18)	0.40422 (13)	0.41439 (5)	0.0182 (3)	
C14B	0.80027 (18)	0.48834 (14)	0.41132 (5)	0.0205 (3)	
H14B	0.7433	0.5056	0.4334	0.025*	
C15B	0.77918 (18)	0.54699 (13)	0.37620 (5)	0.0182 (3)	
H15B	0.7088	0.6028	0.3751	0.022*	
C16B	0.9216 (2)	0.33528 (14)	0.45152 (5)	0.0215 (3)	
H16C	0.8740	0.3733	0.4735	0.026*	
H16D	1.0248	0.3293	0.4579	0.026*	
C17B	0.85697 (19)	0.21766 (13)	0.44763 (5)	0.0195 (3)	
H17B	0.9058	0.1806	0.4253	0.023*	
C18B	0.8867 (2)	0.14987 (15)	0.48509 (5)	0.0313 (4)	
H18D	0.8488	0.0759	0.4817	0.047*	
H18E	0.8399	0.1847	0.5075	0.047*	
H18F	0.9902	0.1463	0.4897	0.047*	
C19B	0.6939 (2)	0.22087 (16)	0.43873 (6)	0.0285 (4)	
H19D	0.6584	0.1464	0.4350	0.043*	
H19E	0.6771	0.2634	0.4149	0.043*	
H19F	0.6432	0.2549	0.4606	0.043*	
C20B	0.76379 (18)	0.69647 (14)	0.30640 (5)	0.0190 (3)	
H20D	0.8130	0.7416	0.3258	0.029*	
H20E	0.6641	0.6853	0.3144	0.029*	
H20F	0.7659	0.7332	0.2810	0.029*	
H1NA	0.568 (2)	0.9602 (19)	0.2475 (6)	0.034 (6)*	
H1NB	0.918 (2)	0.4564 (18)	0.2501 (6)	0.022 (6)*	

### Atomic displacement parameters (Å^2^)

**Table:**

	*U*^11^	*U*^22^	*U*^33^	*U*^12^	*U*^13^	*U*^23^
Br1A	0.02409 (8)	0.02207 (8)	0.03074 (9)	0.00076 (7)	0.00373 (8)	−0.00849 (8)
O1A	0.0144 (5)	0.0287 (6)	0.0207 (5)	0.0038 (5)	0.0014 (5)	−0.0010 (5)
N1A	0.0173 (7)	0.0183 (6)	0.0162 (7)	0.0044 (6)	0.0033 (5)	0.0026 (5)
N2A	0.0131 (6)	0.0205 (6)	0.0155 (6)	0.0042 (6)	0.0016 (5)	−0.0004 (5)
C1A	0.0171 (8)	0.0192 (7)	0.0188 (8)	0.0012 (7)	−0.0003 (7)	0.0027 (6)
C2A	0.0161 (8)	0.0172 (7)	0.0228 (8)	0.0033 (7)	0.0031 (7)	0.0018 (6)
C3A	0.0178 (8)	0.0179 (7)	0.0209 (8)	−0.0027 (7)	0.0042 (7)	−0.0021 (6)
C4A	0.0196 (8)	0.0222 (8)	0.0216 (8)	0.0005 (7)	−0.0010 (7)	−0.0018 (6)
C5A	0.0159 (8)	0.0193 (7)	0.0225 (8)	0.0028 (7)	−0.0008 (7)	0.0008 (6)
C6A	0.0152 (9)	0.0174 (7)	0.0169 (8)	0.0003 (6)	0.0018 (6)	0.0015 (6)
C7A	0.0162 (8)	0.0176 (7)	0.0190 (7)	0.0044 (7)	0.0014 (7)	0.0004 (6)
C8A	0.0170 (7)	0.0194 (8)	0.0146 (7)	0.0024 (7)	0.0025 (6)	0.0047 (6)
C9A	0.0137 (7)	0.0185 (7)	0.0194 (7)	0.0033 (7)	0.0019 (6)	0.0010 (6)
C10A	0.0143 (7)	0.0179 (6)	0.0144 (6)	0.0015 (6)	0.0024 (5)	−0.0011 (5)
C11A	0.0153 (7)	0.0213 (8)	0.0188 (8)	−0.0011 (6)	−0.0001 (6)	−0.0003 (6)
C12A	0.0180 (8)	0.0182 (7)	0.0185 (8)	−0.0042 (6)	0.0003 (6)	−0.0019 (6)
C13A	0.0183 (7)	0.0173 (6)	0.0160 (7)	−0.0005 (6)	0.0027 (6)	−0.0021 (5)
C14A	0.0160 (7)	0.0293 (8)	0.0160 (7)	−0.0025 (7)	−0.0004 (6)	0.0036 (6)
C15A	0.0133 (7)	0.0284 (8)	0.0184 (7)	−0.0060 (7)	−0.0002 (5)	0.0019 (6)
C16A	0.0206 (8)	0.0173 (7)	0.0203 (7)	0.0020 (6)	0.0031 (6)	0.0025 (6)
C17A	0.0228 (8)	0.0232 (8)	0.0197 (8)	0.0028 (7)	0.0051 (6)	0.0041 (6)
C18A	0.0875 (18)	0.0250 (9)	0.0202 (9)	0.0014 (11)	0.0156 (11)	−0.0014 (7)
C19A	0.0312 (10)	0.0280 (9)	0.0231 (8)	−0.0014 (8)	0.0046 (7)	0.0094 (7)
C20A	0.0222 (9)	0.0197 (8)	0.0269 (9)	0.0020 (7)	0.0041 (7)	0.0033 (7)
Br1B	0.03377 (10)	0.01815 (7)	0.02155 (8)	0.00059 (7)	−0.00595 (8)	−0.00540 (7)
O1B	0.0119 (5)	0.0290 (6)	0.0210 (5)	−0.0029 (5)	0.0010 (4)	−0.0038 (5)
N1B	0.0165 (6)	0.0165 (6)	0.0153 (6)	−0.0018 (6)	−0.0010 (5)	−0.0015 (5)
N2B	0.0129 (7)	0.0206 (7)	0.0160 (6)	−0.0039 (6)	0.0007 (5)	−0.0023 (5)
C1B	0.0196 (9)	0.0174 (7)	0.0154 (7)	0.0001 (7)	0.0008 (7)	0.0005 (6)
C2B	0.0186 (8)	0.0163 (6)	0.0200 (7)	0.0002 (7)	−0.0004 (7)	0.0016 (6)
C3B	0.0249 (8)	0.0126 (6)	0.0194 (8)	0.0011 (7)	−0.0037 (7)	−0.0018 (6)
C4B	0.0222 (8)	0.0185 (7)	0.0161 (7)	0.0041 (7)	−0.0001 (7)	−0.0011 (6)
C5B	0.0189 (8)	0.0185 (7)	0.0167 (7)	0.0014 (7)	0.0005 (6)	0.0007 (6)
C6B	0.0171 (9)	0.0157 (7)	0.0166 (8)	0.0021 (6)	−0.0024 (6)	−0.0007 (6)
C7B	0.0171 (8)	0.0192 (7)	0.0163 (7)	0.0002 (7)	−0.0012 (6)	−0.0005 (6)
C8B	0.0167 (7)	0.0160 (7)	0.0132 (7)	−0.0022 (6)	0.0009 (5)	0.0009 (6)
C9B	0.0145 (7)	0.0150 (6)	0.0135 (6)	−0.0022 (6)	0.0000 (6)	0.0007 (5)
C10B	0.0143 (7)	0.0139 (6)	0.0157 (6)	−0.0014 (6)	−0.0014 (5)	−0.0005 (5)
C11B	0.0162 (7)	0.0173 (7)	0.0160 (7)	0.0007 (6)	0.0015 (6)	−0.0022 (6)
C12B	0.0172 (8)	0.0146 (6)	0.0245 (8)	0.0023 (6)	−0.0027 (6)	−0.0005 (6)
C13B	0.0213 (7)	0.0173 (6)	0.0160 (7)	−0.0021 (6)	−0.0030 (6)	0.0005 (5)
C14B	0.0255 (8)	0.0221 (7)	0.0139 (6)	0.0036 (7)	0.0012 (6)	0.0000 (6)
C15B	0.0180 (7)	0.0184 (7)	0.0182 (7)	0.0038 (6)	0.0007 (6)	−0.0006 (6)
C16B	0.0285 (9)	0.0212 (7)	0.0148 (7)	−0.0026 (7)	−0.0066 (6)	0.0023 (6)
C17B	0.0236 (8)	0.0190 (7)	0.0159 (7)	0.0002 (7)	−0.0014 (6)	0.0021 (5)
C18B	0.0449 (12)	0.0244 (8)	0.0246 (9)	−0.0040 (8)	−0.0040 (8)	0.0067 (7)
C19B	0.0244 (9)	0.0293 (9)	0.0318 (9)	−0.0033 (7)	0.0007 (8)	0.0004 (8)
C20B	0.0224 (8)	0.0166 (7)	0.0180 (7)	0.0015 (6)	−0.0013 (6)	0.0018 (6)

### Geometric parameters (Å, °)

**Table:**

Br1A—C3A	1.8948 (17)	Br1B—C3B	1.8944 (16)
O1A—C8A	1.2304 (19)	O1B—C8B	1.2349 (18)
N1A—C7A	1.286 (2)	N1B—C7B	1.282 (2)
N1A—N2A	1.3830 (18)	N1B—N2B	1.3868 (18)
N2A—C8A	1.348 (2)	N2B—C8B	1.345 (2)
N2A—H1NA	0.91 (2)	N2B—H1NB	0.770 (19)
C1A—C2A	1.383 (2)	C1B—C2B	1.389 (2)
C1A—C6A	1.405 (3)	C1B—C6B	1.393 (3)
C1A—H1AA	0.93	C1B—H1BA	0.93
C2A—C3A	1.387 (2)	C2B—C3B	1.396 (2)
C2A—H2AA	0.93	C2B—H2BA	0.93
C3A—C4A	1.383 (2)	C3B—C4B	1.386 (2)
C4A—C5A	1.390 (2)	C4B—C5B	1.387 (2)
C4A—H4AA	0.93	C4B—H4BA	0.93
C5A—C6A	1.394 (2)	C5B—C6B	1.403 (2)
C5A—H5AA	0.93	C5B—H5BA	0.93
C6A—C7A	1.463 (2)	C6B—C7B	1.468 (2)
C7A—H7AA	0.93	C7B—H7BA	0.93
C8A—C9A	1.533 (2)	C8B—C9B	1.530 (2)
C9A—C10A	1.526 (2)	C9B—C10B	1.526 (2)
C9A—C20A	1.532 (2)	C9B—C20B	1.531 (2)
C9A—H9AA	0.98	C9B—H9BA	0.98
C10A—C15A	1.393 (2)	C10B—C15B	1.394 (2)
C10A—C11A	1.396 (2)	C10B—C11B	1.400 (2)
C11A—C12A	1.388 (2)	C11B—C12B	1.385 (2)
C11A—H11A	0.93	C11B—H11B	0.93
C12A—C13A	1.394 (2)	C12B—C13B	1.392 (2)
C12A—H12A	0.93	C12B—H12B	0.93
C13A—C14A	1.392 (2)	C13B—C14B	1.393 (2)
C13A—C16A	1.507 (2)	C13B—C16B	1.505 (2)
C14A—C15A	1.390 (2)	C14B—C15B	1.387 (2)
C14A—H14A	0.93	C14B—H14B	0.93
C15A—H15A	0.93	C15B—H15B	0.93
C16A—C17A	1.533 (2)	C16B—C17B	1.537 (2)
C16A—H16A	0.97	C16B—H16C	0.97
C16A—H16B	0.97	C16B—H16D	0.97
C17A—C18A	1.505 (3)	C17B—C19B	1.522 (3)
C17A—C19A	1.532 (2)	C17B—C18B	1.523 (2)
C17A—H17A	0.98	C17B—H17B	0.98
C18A—H18A	0.96	C18B—H18D	0.96
C18A—H18B	0.96	C18B—H18E	0.96
C18A—H18C	0.96	C18B—H18F	0.96
C19A—H19A	0.96	C19B—H19D	0.96
C19A—H19B	0.96	C19B—H19E	0.96
C19A—H19C	0.96	C19B—H19F	0.96
C20A—H20A	0.96	C20B—H20D	0.96
C20A—H20B	0.96	C20B—H20E	0.96
C20A—H20C	0.96	C20B—H20F	0.96
			
C7A—N1A—N2A	114.27 (13)	C7B—N1B—N2B	114.15 (13)
C8A—N2A—N1A	120.01 (13)	C8B—N2B—N1B	120.36 (13)
C8A—N2A—H1NA	121.9 (14)	C8B—N2B—H1NB	118.0 (16)
N1A—N2A—H1NA	118.1 (14)	N1B—N2B—H1NB	121.7 (16)
C2A—C1A—C6A	120.33 (16)	C2B—C1B—C6B	120.66 (15)
C2A—C1A—H1AA	119.8	C2B—C1B—H1BA	119.7
C6A—C1A—H1AA	119.8	C6B—C1B—H1BA	119.7
C1A—C2A—C3A	119.30 (15)	C1B—C2B—C3B	118.66 (16)
C1A—C2A—H2AA	120.3	C1B—C2B—H2BA	120.7
C3A—C2A—H2AA	120.3	C3B—C2B—H2BA	120.7
C4A—C3A—C2A	121.77 (15)	C4B—C3B—C2B	121.92 (15)
C4A—C3A—Br1A	118.38 (13)	C4B—C3B—Br1B	118.22 (12)
C2A—C3A—Br1A	119.85 (13)	C2B—C3B—Br1B	119.86 (13)
C3A—C4A—C5A	118.49 (16)	C3B—C4B—C5B	118.57 (15)
C3A—C4A—H4AA	120.8	C3B—C4B—H4BA	120.7
C5A—C4A—H4AA	120.8	C5B—C4B—H4BA	120.7
C4A—C5A—C6A	121.17 (16)	C4B—C5B—C6B	120.84 (16)
C4A—C5A—H5AA	119.4	C4B—C5B—H5BA	119.6
C6A—C5A—H5AA	119.4	C6B—C5B—H5BA	119.6
C5A—C6A—C1A	118.87 (15)	C1B—C6B—C5B	119.29 (15)
C5A—C6A—C7A	118.81 (16)	C1B—C6B—C7B	122.62 (15)
C1A—C6A—C7A	122.32 (15)	C5B—C6B—C7B	118.06 (16)
N1A—C7A—C6A	120.87 (16)	N1B—C7B—C6B	120.80 (16)
N1A—C7A—H7AA	119.6	N1B—C7B—H7BA	119.6
C6A—C7A—H7AA	119.6	C6B—C7B—H7BA	119.6
O1A—C8A—N2A	124.37 (16)	O1B—C8B—N2B	123.79 (15)
O1A—C8A—C9A	121.71 (15)	O1B—C8B—C9B	121.70 (15)
N2A—C8A—C9A	113.78 (13)	N2B—C8B—C9B	114.50 (13)
C10A—C9A—C20A	114.55 (13)	C10B—C9B—C8B	107.11 (12)
C10A—C9A—C8A	106.07 (12)	C10B—C9B—C20B	114.91 (13)
C20A—C9A—C8A	110.26 (14)	C8B—C9B—C20B	109.92 (13)
C10A—C9A—H9AA	108.6	C10B—C9B—H9BA	108.2
C20A—C9A—H9AA	108.6	C8B—C9B—H9BA	108.2
C8A—C9A—H9AA	108.6	C20B—C9B—H9BA	108.2
C15A—C10A—C11A	118.25 (14)	C15B—C10B—C11B	118.15 (14)
C15A—C10A—C9A	121.57 (14)	C15B—C10B—C9B	122.14 (13)
C11A—C10A—C9A	120.10 (14)	C11B—C10B—C9B	119.70 (13)
C12A—C11A—C10A	120.65 (15)	C12B—C11B—C10B	120.68 (15)
C12A—C11A—H11A	119.7	C12B—C11B—H11B	119.7
C10A—C11A—H11A	119.7	C10B—C11B—H11B	119.7
C11A—C12A—C13A	121.41 (15)	C11B—C12B—C13B	121.51 (15)
C11A—C12A—H12A	119.3	C11B—C12B—H12B	119.2
C13A—C12A—H12A	119.3	C13B—C12B—H12B	119.2
C14A—C13A—C12A	117.58 (14)	C12B—C13B—C14B	117.42 (14)
C14A—C13A—C16A	121.26 (15)	C12B—C13B—C16B	120.39 (15)
C12A—C13A—C16A	121.16 (15)	C14B—C13B—C16B	122.10 (15)
C15A—C14A—C13A	121.43 (15)	C15B—C14B—C13B	121.77 (15)
C15A—C14A—H14A	119.3	C15B—C14B—H14B	119.1
C13A—C14A—H14A	119.3	C13B—C14B—H14B	119.1
C14A—C15A—C10A	120.65 (15)	C14B—C15B—C10B	120.47 (15)
C14A—C15A—H15A	119.7	C14B—C15B—H15B	119.8
C10A—C15A—H15A	119.7	C10B—C15B—H15B	119.8
C13A—C16A—C17A	116.02 (13)	C13B—C16B—C17B	113.29 (13)
C13A—C16A—H16A	108.3	C13B—C16B—H16C	108.9
C17A—C16A—H16A	108.3	C17B—C16B—H16C	108.9
C13A—C16A—H16B	108.3	C13B—C16B—H16D	108.9
C17A—C16A—H16B	108.3	C17B—C16B—H16D	108.9
H16A—C16A—H16B	107.4	H16C—C16B—H16D	107.7
C18A—C17A—C19A	110.80 (15)	C19B—C17B—C18B	110.54 (15)
C18A—C17A—C16A	112.47 (16)	C19B—C17B—C16B	111.74 (15)
C19A—C17A—C16A	109.00 (14)	C18B—C17B—C16B	110.65 (13)
C18A—C17A—H17A	108.1	C19B—C17B—H17B	107.9
C19A—C17A—H17A	108.1	C18B—C17B—H17B	107.9
C16A—C17A—H17A	108.1	C16B—C17B—H17B	107.9
C17A—C18A—H18A	109.5	C17B—C18B—H18D	109.5
C17A—C18A—H18B	109.5	C17B—C18B—H18E	109.5
H18A—C18A—H18B	109.5	H18D—C18B—H18E	109.5
C17A—C18A—H18C	109.5	C17B—C18B—H18F	109.5
H18A—C18A—H18C	109.5	H18D—C18B—H18F	109.5
H18B—C18A—H18C	109.5	H18E—C18B—H18F	109.5
C17A—C19A—H19A	109.5	C17B—C19B—H19D	109.5
C17A—C19A—H19B	109.5	C17B—C19B—H19E	109.5
H19A—C19A—H19B	109.5	H19D—C19B—H19E	109.5
C17A—C19A—H19C	109.5	C17B—C19B—H19F	109.5
H19A—C19A—H19C	109.5	H19D—C19B—H19F	109.5
H19B—C19A—H19C	109.5	H19E—C19B—H19F	109.5
C9A—C20A—H20A	109.5	C9B—C20B—H20D	109.5
C9A—C20A—H20B	109.5	C9B—C20B—H20E	109.5
H20A—C20A—H20B	109.5	H20D—C20B—H20E	109.5
C9A—C20A—H20C	109.5	C9B—C20B—H20F	109.5
H20A—C20A—H20C	109.5	H20D—C20B—H20F	109.5
H20B—C20A—H20C	109.5	H20E—C20B—H20F	109.5
			
C7A—N1A—N2A—C8A	−172.90 (15)	C7B—N1B—N2B—C8B	171.64 (15)
C6A—C1A—C2A—C3A	1.7 (3)	C6B—C1B—C2B—C3B	−0.1 (2)
C1A—C2A—C3A—C4A	−2.0 (3)	C1B—C2B—C3B—C4B	1.5 (2)
C1A—C2A—C3A—Br1A	178.62 (12)	C1B—C2B—C3B—Br1B	−178.25 (12)
C2A—C3A—C4A—C5A	0.3 (3)	C2B—C3B—C4B—C5B	−0.9 (2)
Br1A—C3A—C4A—C5A	179.62 (13)	Br1B—C3B—C4B—C5B	178.82 (12)
C3A—C4A—C5A—C6A	1.8 (3)	C3B—C4B—C5B—C6B	−1.0 (2)
C4A—C5A—C6A—C1A	−2.1 (3)	C2B—C1B—C6B—C5B	−1.7 (2)
C4A—C5A—C6A—C7A	177.39 (16)	C2B—C1B—C6B—C7B	176.39 (15)
C2A—C1A—C6A—C5A	0.3 (3)	C4B—C5B—C6B—C1B	2.3 (2)
C2A—C1A—C6A—C7A	−179.19 (15)	C4B—C5B—C6B—C7B	−175.90 (14)
N2A—N1A—C7A—C6A	−176.44 (13)	N2B—N1B—C7B—C6B	179.61 (13)
C5A—C6A—C7A—N1A	−155.46 (16)	C1B—C6B—C7B—N1B	−20.0 (2)
C1A—C6A—C7A—N1A	24.0 (2)	C5B—C6B—C7B—N1B	158.13 (15)
N1A—N2A—C8A—O1A	5.2 (3)	N1B—N2B—C8B—O1B	−4.2 (3)
N1A—N2A—C8A—C9A	−170.40 (13)	N1B—N2B—C8B—C9B	174.74 (13)
O1A—C8A—C9A—C10A	−82.77 (19)	O1B—C8B—C9B—C10B	82.66 (19)
N2A—C8A—C9A—C10A	93.00 (16)	N2B—C8B—C9B—C10B	−96.26 (16)
O1A—C8A—C9A—C20A	41.8 (2)	O1B—C8B—C9B—C20B	−42.8 (2)
N2A—C8A—C9A—C20A	−142.45 (15)	N2B—C8B—C9B—C20B	138.28 (14)
C8A—C9A—C10A—C11A	−96.54 (17)	C8B—C9B—C10B—C11B	77.53 (17)
C8A—C9A—C10A—C15A	80.18 (18)	C8B—C9B—C10B—C15B	−101.83 (16)
C20A—C9A—C10A—C11A	141.61 (15)	C20B—C9B—C10B—C11B	−160.06 (14)
C20A—C9A—C10A—C15A	−41.7 (2)	C20B—C9B—C10B—C15B	20.6 (2)
C15A—C10A—C11A—C12A	−0.6 (2)	C15B—C10B—C11B—C12B	0.4 (2)
C9A—C10A—C11A—C12A	176.19 (15)	C9B—C10B—C11B—C12B	−178.97 (15)
C10A—C11A—C12A—C13A	−1.1 (2)	C10B—C11B—C12B—C13B	0.4 (2)
C11A—C12A—C13A—C14A	1.9 (2)	C11B—C12B—C13B—C14B	−0.9 (2)
C11A—C12A—C13A—C16A	−178.41 (14)	C11B—C12B—C13B—C16B	175.84 (15)
C12A—C13A—C14A—C15A	−1.0 (2)	C12B—C13B—C14B—C15B	0.6 (2)
C16A—C13A—C14A—C15A	179.36 (15)	C16B—C13B—C14B—C15B	−176.03 (15)
C13A—C14A—C15A—C10A	−0.8 (3)	C13B—C14B—C15B—C10B	0.1 (3)
C11A—C10A—C15A—C14A	1.6 (2)	C11B—C10B—C15B—C14B	−0.7 (2)
C9A—C10A—C15A—C14A	−175.20 (15)	C9B—C10B—C15B—C14B	178.71 (15)
C14A—C13A—C16A—C17A	103.66 (19)	C12B—C13B—C16B—C17B	−72.7 (2)
C12A—C13A—C16A—C17A	−76.0 (2)	C14B—C13B—C16B—C17B	103.90 (18)
C13A—C16A—C17A—C18A	−53.4 (2)	C13B—C16B—C17B—C19B	−60.1 (2)
C13A—C16A—C17A—C19A	−176.68 (15)	C13B—C16B—C17B—C18B	176.24 (16)

### Hydrogen-bond geometry (Å, °)

**Table:**

*D*—H···*A*	*D*—H	H···*A*	*D*···*A*	*D*—H···*A*
N2A—H1NA···O1B^i^	0.91 (2)	1.97 (2)	2.830 (2)	157 (2)
N2B—H1NB···O1A^ii^	0.77 (2)	2.11 (2)	2.826 (2)	155 (2)
C7A—H7AA···O1B^i^	0.93	2.45	3.241 (2)	143
C7B—H7BA···O1A^ii^	0.93	2.53	3.307 (3)	141
C20B—H20F···N1A	0.96	2.56	3.494 (2)	164
C12A—H12A···Cg2^i^	0.93	2.66	3.482 (2)	148
C12B—H12B···Cg1^ii^	0.93	2.79	3.680 (2)	160

Symmetry codes: (i) −*x*+1, *y*+1/2, −*z*+1/2; (ii) −*x*+2, *y*−1/2, −*z*+1/2.
